# Does a reduction of vascular relaxation promote hypertension?

**DOI:** 10.1186/1471-2210-11-S1-P64

**Published:** 2011-08-01

**Authors:** Kathrin Schulte, Doris Koesling, Evanthia Mergia

**Affiliations:** 1 Department of Pharmacology and Toxicology, Ruhr-Universität-Bochum, Germany

## Background

Alterations in vascular relaxation have been reported in various models of hypertension. With its tonic activity, the NO/cGMP signalling cascade plays an important role in the regulation of the vascular tone. In blood vessels, NO is continuously being produced by the endothelial NO synthases (eNOS) which leads to cGMP production by stimulating the NO-sensitive guanylyl cyclases (NO-GCs). In vascular smooth muscle cells, two NO-GC isoforms, NO-GC1 and NO-GC2, mediate relaxation, with NO-GC1 being responsible for approximately 90% of the NO-stimulated cGMP formation. Deletion of eNOS or both NO-GCs abrogates endothelium-dependent relaxation and causes substantial hypertension. However, deletion of only NO-GC1 does not lead to hypertension despite a 50% reduction of endothelium-dependent relaxation.

## Results

To investigate whether reduced vascular relaxation promotes the development of hypertension, we induced hypertension in NO-GC1 KO and wild-type (WT) mice by treating the mice with angiotensin II treatment (1.44 mg / kg BW / d) for two weeks. As expected, angiotensin II treatment induced profound hypertension. However, blood pressure increases (~40 mmHg) and reduction in endothelium-dependent relaxation were similar in KO and WT mice. Also angiotensin II-induced increases in total peripheral resistance as measured in hind-limb perfusion experiments were indistinguishable. Interestingly, NO-GC1 KO mice displayed 35% higher basal peripheral resistance in these experiments.

## Conclusion

Taken together, reduction of NO-stimulated cGMP production in blood vessels by 90% does not exaggerate angiotensin II-induced hypertension. The mechanisms underlying this finding have to be investigated.

